# Anti-Inflammatory Activity of Biotransformed *Platycodon grandiflorum* Root Extracts Containing 3-*O*-β-D-Glucopyranosyl Platycosides in LPS-Stimulated Alveolar Macrophages, NR8383 Cells

**DOI:** 10.4014/jmb.2408.08005

**Published:** 2024-10-31

**Authors:** Jeong Won Choi, Hyeok Jin Choi, Chae Sun Na, Hwan Lee, Byung Joo Lee, Kyung-Chul Shin, Jin Boo Jeong

**Affiliations:** 1 Department of Forest Science, Andong National University, Andong 36729, Republic of Korea; 2 Division of Wild Plant and Seeds, Baekdudaegan National Arboretum, Bonghwa 36029, Republic of Korea; 3 Lifeceutical Bio Food Research Center, Seoul 06138, Republic of Korea; 4 Department of Bioscience and Biotechnology, Hankuk University of Foreign Studies, Yongin 17035, Republic of Korea

**Keywords:** *Platycodon grandiflorum*, anti-inflammatory activity, alveolar macrophage, deglycosylation

## Abstract

Acute lung injury (ALI) is a severe inflammatory condition characterized by excessive immune responses and oxidative stress, leading to significant tissue damage. Given the need for novel therapeutic agents, this study aimed to explore the anti-inflammatory activity and mechanisms of biotransformed *Platycodon grandiflorum* root extracts (BT-PGR), which were enzymatically processed using rapidsase PL Classic from *Aspergillus niger*. The goal was to assess the potential of BT-PGR as a natural treatment for ALI. BT-PGR effectively inhibited the production of NO, iNOS, IL-1β, IL-6, and TNF-α induced by LPS in NR8383 cells. BT-PGR inhibited the phosphorylation of ERK1/2, p38, JNK and p65 in LPS-stimulated NR8383 cells. In addition, BT-PGR suppressed LPS-mediated activation of NF-κB luciferase activity. BT-PGR increased the levels of HO-1 and the inhibition of HO-1 by ZnPP attenuated BT-PGR-mediated inhibition of NO production. In addition, the inhibition of PI3K by LY294002 blocked the BT-PGR-mediated increase of HO-1 level. BT-PGR increased nuclear Nrf2 level and the knockdown of Nrf2 by siRNA inhibited BT-PGR-mediated increase of HO-1 level. In addition, inhibition of PI3K by LY294002 suppressed the increase of nuclear Nrf2 level. Based on these results, it can be inferred that BT-PGR exhibits anti-inflammatory activity in rat alveolar macrophages, suggesting its potential as a natural candidate for the improvement of ALI.

## Introduction

Acute lung injury (ALI), characterized by diffuse alveolar injury, is a critical condition prevalent among patients in intensive care, often resulting in elevated rates of morbidity and mortality [1]. The unregulated acute inflammatory response, along with the excessive secretion of pro-inflammatory cytokines, is regarded as two pivotal pathological contributors to the onset of ALI [2]. It has been reported that macrophages, neutrophils, lymphocytes, pulmonary epithelial fibroblasts, and platelets are implicated in the development and progression of ALI [3, 4]. In particular, the hyperactive inflammatory response of alveolar macrophages is considered a major pathological factor in the initiation of ALI [5]. Consequently, attenuating the inflammatory response of alveolar macrophages and regulating the production of inflammatory mediators secreted by them are increasingly considered pivotal strategies for the treatment of ALI [5].

*Platycodon grandiflorum* (*P. grandiflorum*) belonging to the Campanulaceae family is a perennial herb native to Northeast Asia. *P. grandiflorum* root (PGR) has been used as side dishes, desserts, teas, and specialty liquors. Additionally, PGR is extensively utilized as dietary supplements, particularly for managing pulmonary ailments and respiratory conditions [6]. Moreover, the saponins present in PGR, known as platycosides, have been reported to exhibit a spectrum of pharmacological activities. These include anti-obesity, anti-inflammatory, anti-allergic, antioxidative, neuroprotective, and anti-cancer properties [6] Saponins within PGR are primarily glycosylated saponins with more than three sugar moieties, known for their limited absorption in the intestine. Conversely, saponins with fewer than two sugar residues, referred to as deglycosylated saponins, are recognized for their enhanced absorption from the gastrointestinal tract into the bloodstream [6]. In addition, Saponins that have undergone deglycosylation, a process transforming glycosylated saponins, demonstrate enhanced anti-inflammatory activity compared to their glycosylated counterparts [6]. However, research into the anti-inflammatory mechanisms of action associated with the deglycosylated saponins of PGR remains insufficiently explored. Consequently, the present study aimed to evaluate the anti-inflammatory activity of deglycosylated PGR extracts, facilitated using the enzyme rapidsase PL Classic derived from *Aspergillus niger*.

## Materials and Methods

### Materials

PGR was purchased from a local market (Republic of Korea). Platycoside standards, including platycoside E (PE), platycodin D_3_ (PD_3_), platycodin D (PD), deapiosylated platycodin D_3_ (deapi-PD_3_), deapiosylated platycodin D (deapi-PD), polygalacin D_3_ (PGD_3_), polygalacin D (PGD), and platyconic acid A (PCAA) were purchased from Ambo Institute (Republic of Korea), while glucosyl-platycodigenin (glc-PDN), glucosyl-polygalacic acid (glc-PGA), and glucosyl-platyconic acid (glc-PCA) were prepared by purifying the products obtained from the conversions of PE, PGD, and PCAA using Pectinex Ultra SP-L. Rapidase PL Classic was provided from DKSH (Switzerland). The reagents MTT, PD98059, SB203580, SP600125, BAY 11-7082, LY294002, Griess reagent, and LPS were procured from Sigma-Aldrich (USA). Zinc (II) Protoporphyrin IX (ZnPP) was purchased from Enzo Life Sciences (USA). The primary antibodies such as p-ERK1/2, ERK1/2, p-p38, p38, p-JNK, JNK, p-p65, p65, p-PI3K, PI3K, HO-1, Nrf2, and β-actin were purchased from Cell Signaling Technology (USA). The secondary antibody for anti-rabbit IgG, HRP-linked antibody was purchased from Cell Signaling Technology. Control- and Nrf2-siRNA were purchased from Cell Signaling Technology and Santa Cruz Biotechnology (USA).

### Preparation of Biotransformed PGR

Washed, peeled, dried, and sliced PGR (5-year-old) weighing 20 kg were subjected to an extraction process. Purified water, amounting to 20 times the weight of the PGR, was added. The mixture was then subjected to a vacuum-assisted circulatory extraction at a temperature range of 60−80°C for approximately 10-12 h. Following the extraction process, the cooled, clear extract solution was subjected to sugar removal using UBK530 resin. The resulting sugar-depleted extract was used as PGR extract for further biotransformation process. The biotransformation of PGR extract was performed at 55°C in 50 mM citrate-phosphate buffer (pH 5.5) containing 1.5 mg/ml Rapidase PL Classic and 10% (w/v) PGR extract for 18 h. The enzyme was inactivated with incubation at 100°C for 1 h, and it was filtered using 5 micron and 1 micron sediment filters. Following the filtration, the processed PGR extract was concentrated to a level below 20 brix. This concentrated extract (BT-PGR, Solid content: 19%) was then meticulously stored at a refrigerated temperature range of 3°C to 5°C.

### Cell Line and Cell Culture

In this study, the rat alveolar macrophage cell line, NR8383, was employed due to its prevalent application in screening agents with inhibitory activity against ALI in cellular models [5]. The NR8383 cells, acquired from the American Type Culture Collection (USA), were meticulously maintained in a Ham's F-12K medium. This medium was strategically supplemented with 15% fetal bovine serum to provide essential nutrients and growth factors. Additionally, to ensure an optimal and contamination-free culture environment, the medium was enriched with 100 μg/ml streptomycin and 100 U/ml penicillin, serving as crucial antimicrobial agents. These cells were incubated at a controlled temperature of 37°C, within a humidity-regulated environment maintaining a 5% CO_2_ atmosphere.

### Griess Assay

The inhibitory activity of BT-PGR on nitric oxide (NO) production in NR8383 cells stimulated with LPS was validated using the Griess assay. The cells were cultured in a 96-well plate for 24 h, following which they were pre-treated with BT-PGR for 2 h prior to the addition of LPS at a concentration of 1 μg/ml for an 18-h incubation period. Subsequent to this treatment, the cell culture supernatant was mixed in a 1:1 ratio with Griess reagent and then allowed to stand at room temperature for 15 min. The absorbance of this mixture was subsequently measured at a wavelength of 540 nm using a UV/Visible spectrophotometer (Human Cop., Xma-3000PC, Republic of Korea).

### ELISA Assay

The inhibitory activities of BT-PGR on the production of interleukin-1β (IL-1β), interleukin-6 (IL-6), and tumor necrosis factor-α (TNF-α) in LPS-stimulated NR8383 cells were evaluated using IL-1β Mouse ELISA Kit (Invitrogen, USA), IL-6 Mouse ELISA Kit (Invitrogen), and TNF-α Mouse ELISA Kit (Invitrogen), respectively. The cells were cultured in a 96-well plate for 24 h, following which they were pre-treated with BT-PGR for 2 h prior to the addition of LPS at a concentration of 1 μg/ml for an 18-h incubation period. Subsequent to this treatment, the cell culture supernatant was utilized for ELISA analysis, strictly adhering to the protocols provided by the manufacturer.

### Reverse Transcription Polymerase Chain Reaction (RT-PCR)

Upon the completion of the experimental treatments, total RNA extraction was conducted from the cell samples using a RNeasy Mini Kit (Qiagen, USA). This was followed by a quantitative assessment of the extracted RNA. For cDNA synthesis, 1 μg of the total RNA was utilized, employing a Verso cDNA Kit (Thermo Fisher Scientific, USA) for the process. PCR amplifications were carried out with a PCR Master Mix Kit (Promega, USA), using specific primers as listed in Table 1. Post-PCR, the amplification products were analyzed through agarose gel electrophoresis to allow for their visual inspection. The mRNA bands were quantitatively assessed for their intensity using the UN-SCAN-IT gel software version 5.1 (Silk Scientific Inc., USA).

### Isolation of Nuclear Protein from NR8383 Cells

After all experimental treatments were concluded, nuclear proteins were extracted from NR8383 cells utilizing an Active Motif Nuclear Extract Kit (ACTIVE MOTIF, USA). This extraction was performed in strict accordance with the guidelines provided by the manufacturer.

### Transfection of Small Interference RNA

NR8383 cells were seeded into 6-well plates and allowed to incubate overnight. Following this, the cells underwent transfection with both control- and Nrf2-small interference RNAs (siRNAs) at a 100 nM concentration. This transfection process was conducted for 48 h utilizing the TransIT-TKO transfection reagent (Mirus, USA), strictly adhering to the guidelines provided by the manufacturer.

### SDS-PAGE and Western Blot Analysis

Subsequent to completing the treatment, a thorough quantitative protein analysis was carried out using a bicinchoninic acid protein assay kit (Thermo Fisher Scientific). The proteins were then separated via sodium dodecyl sulfate-polyacrylamide gel electrophoresis (SDS-PAGE) and subsequently transferred to nitrocellulose membranes (Thermo Fisher Scientific). These membranes underwent a blocking step for 1 h at ambient temperature, followed by overnight incubation with primary antibodies at 4°C. This was succeeded by a one-hour incubation with secondary antibodies at room temperature. After treating with ECL western blotting substrate, visualization of the protein bands was accomplished using an LI-COR C-DiGit Blot Scanner (LI-COR Biosciences, USA). The intensity of these protein bands was quantitatively determined using the UN-SCAN-IT gel software version 5.1 (Silk Scientific Inc.).

### NF-κB Luciferase Activity

For the analysis of NF-κB luciferase activity in NR8383 cells, transient transfection was carried out using PolyJet DNA Transfection Reagent (SignaGen LaboratoriesUSA). NR8383 cells were plated at a concentration of 1 × 10^5^ cells/well in 12-well plates and incubated for 24 h. Following this incubation, the cells were exposed to plasmid solutions containing 1 μg of NF-κB luciferase reporter plasmids (Addgene, USA) and 0.1 μg of pRL-null vectors. This was succeeded by a further 24-h culture period. The NR8383 cells were then pre-treated with BT-PGR for 2 h before co-treatment with LPS (1 μg/ml for) an additional 24 h. After these treatments, cell lysates were prepared using 1× luciferase lysis buffer. Finally, the luciferase assay was conducted using a dual-luciferase reporter assay system (Promega), with the results normalized to the pRL-null luciferase activity, providing an accurate measure of NF-κB activation.

### Statistical Analysis

All experiments were repeated at least three times. Statistical analyses were verified using GraphPad Prism version 5.0 (Dotmatics) and data are presented as mean ± standard deviation. Each data point was analyzed using one-way analysis of variance and the data was analyzed using the Bonferroni post hoc test. *P* < 0.05 was considered to indicate a statistically significant difference.

### HPLC Analysis

To extract platycosides, *n*-butanol was added to the biotransformed PGR at a ratio of 1:1. The *n*-butanol fraction of the extract was evaporated and then the dried residue was dissolved in methanol. The concentrations of platycosides were determined using the HPLC system (Agilent 1100, USA) equipped with a Hydrosphere C18 column (4.6 × 150 mm, 5 μm particle size, YMC, Japan) at an absorbance of 203 nm. The column was eluted at a flow rate of 1 ml/min at 30°C with a gradient of acetonitrile ranging from 10 to 40% (v/v) for 30 min, 40 to 90% for 15 min, 90 to 10% for 5 min, and constant at 10% for 10 min. All platycosides were quantified using the calibration curves of peak areas with respect to standard solutions of 0.2 to 0.8 mg/ml platycosides.

## Results and Discussion

### Biotransfromation of Platycosides in PGR Using Rapidase PL Classic

**Fig. 1:**
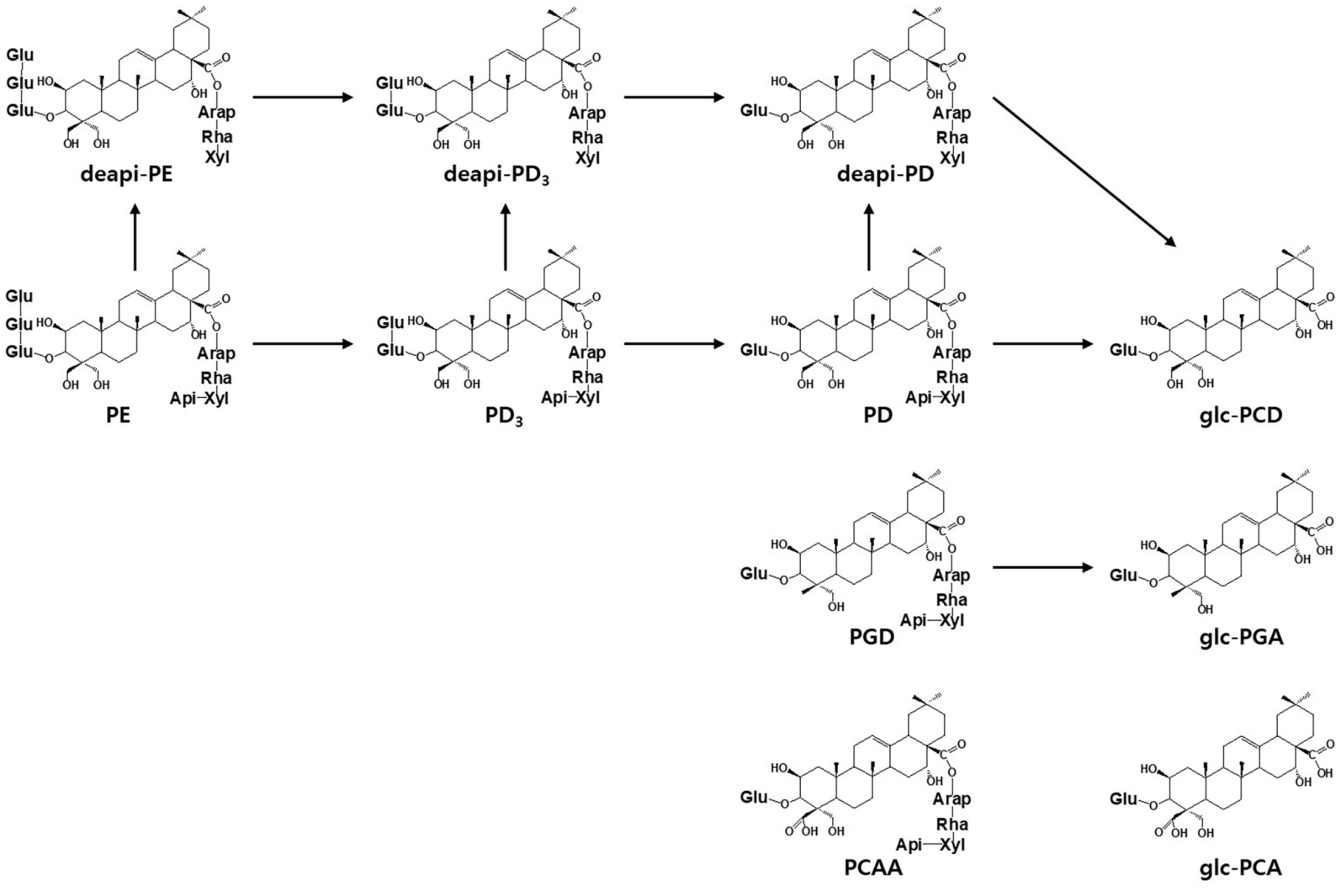
Hydrolytic pathway in the biotransformation of platycosides in PGR extract. (**A**) Pathway of platycodigenintype platycosides, including PE, PD_3_, PD, deapi-PD_3_, deapi-PD, and glc-PDN. (**B**) Pathway of polygalacic acid-type platycosides, including PGD_3_, PGD, and glc-PGA. (**C**) Pathway of polygalacic acid-type platycosides, including PCAA, and glc-PCA. Arap, α-L-arabinopyranosyl-; Rham, α-L-rhamnopyranosyl-; Xyl, β-D-xylopyranosyl-; Api, β-D-apiofuranosyl-; and Glu, β-Dglucopyranosyl-.

The total concentration of platycosides in 5% (w/v) PGR extract was 3.44 mg/ml, while the concentrations of PE, PD_3_, PD, deapi-PE, deapi-PD, PGD, and PCAA were 1.36, 0.11, 0.69, 0.44, 0.04, 0.65, and 0.15 mg/ml (Table 2). After biotransformation, platycosides containing only one glucose such as glc-PCD (0.44 mg/ml), glc-PGA (0.08 mg/ml), and glc-PCA (0.04) (mg/ml) were newly produced from the platycodigenin-type platycosides PE, PD_3_, PD, deapi-PE, deapi-PD; the polygalacic acid-type platycoside PGD; and the platyconic acid-type platycoside PCAA. Based on HPLC analysis, the hydrolytic pathways of the three typical platycoside types were determined, as shown in Fig. 1.

### Effect of BT-PGR on the Production of Inflammatory Mediators in LPS-Stimulated NR8383 Cells

**Fig. 2:**
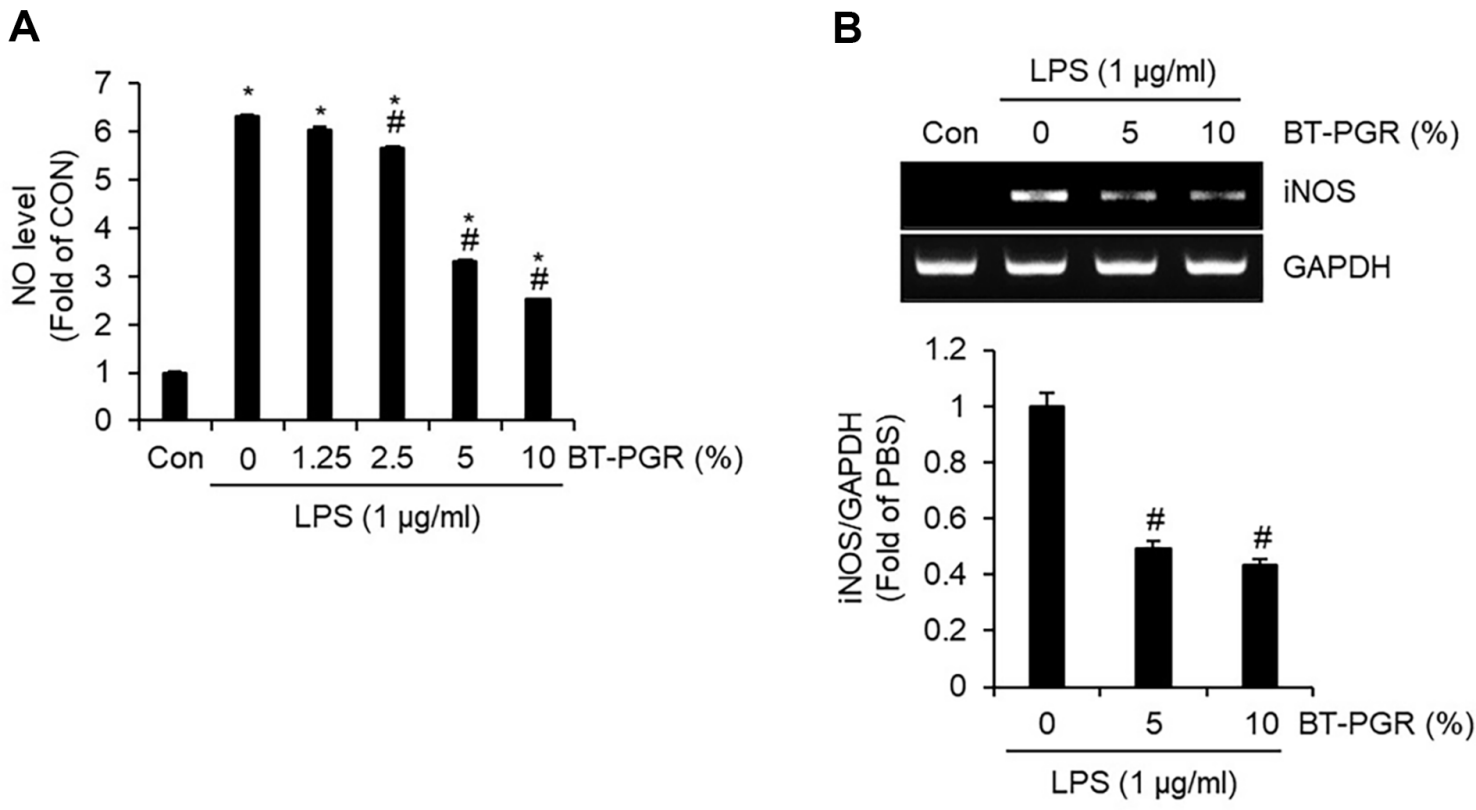
Effect of BT-PGR on NO production and iNOS expression in LPS-stimulated NR8383 cells. NR8383 cells were pretreated with BT-PGR for 2 h and then co-treated with LPS for 18 h. (**A**) NO level was determined by Griess assay. (**B**) iNOS expression was determined by RT-PCR. *P < 0.05 vs. Con group, ^#^*P* < 0.05 vs. PBS group.

**Fig. 3:**
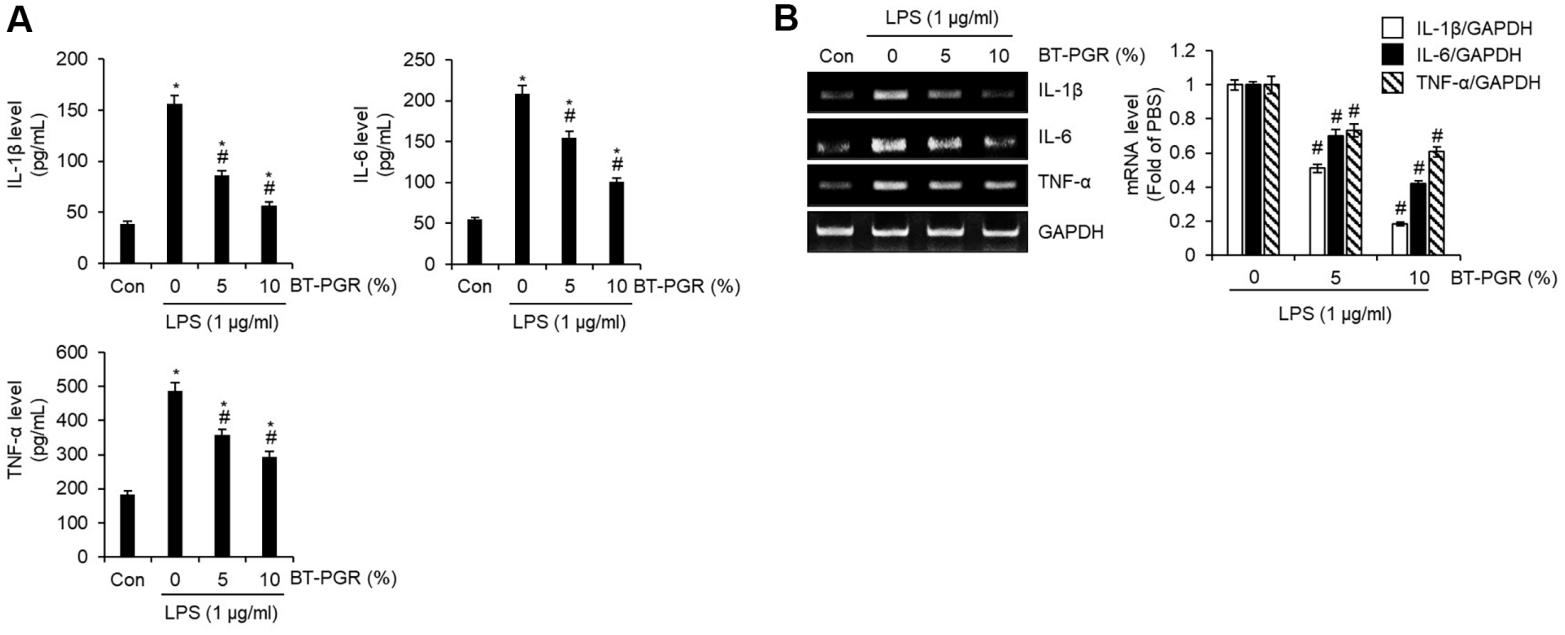
Effect of BT-PGR on the production of inflammatory cytokines in LPS-stimulated NR8383 cells. NR8383 cells were pretreated with BT-PGR for 2 h and then co-treated with LPS for 18 h. (**A**) The levels of IL-1β, IL-6, and TNF- α were determined by ELISA Kit. (**B**) The expression of IL-1β, IL-6, and TNF-α were determined by RT-PCR. *P < 0.05 vs. Con group, ^#^*P* < 0.05 vs. PBS group.

ALI is a prevalent critical disease in clinical settings, often triggered by factors such as infection, trauma, and shock. In its initial stages, ALI is characterized by widespread damage to the alveolar structures and a compromised barrier function in alveolar epithelial cells [7]. The aberrant acute inflammatory response and the resultant overproduction of inflammatory mediators are increasingly recognized as one of the primary pathogenic factors in the onset of ALI [2]. LPS, a component of the cell wall of Gram-negative bacteria, is widely used to induce ALI in cell and animal models due to its potent ability to stimulate the production of inflammatory mediators [8, 9]. Therefore, to evaluate the potential of BT-PGR in preventing ALI, we analyzed its ability to inhibit the overproduction of inflammatory mediators such as NO, inducible nitric oxide synthase (iNOS), IL-1β, IL-6, and TNF-α in NR8383 cells stimulated with LPS. As depicted in Fig. 2A, cells treated solely with LPS (PBS group) exhibited excessive production of nitric oxide (NO) when compared to the untreated cells (Con group). However, upon treatment of the cells with BT-PGR at concentrations ranging from 1.25% to 10% (v/v), it was observed that 5% and 10% BT-PGR treatments notably reduced the LPS-induced overproduction of NO. Furthermore, treatments with 5% and 10% BT-PGR also effectively suppressed the overexpression of iNOS induced by LPS (Fig. 2B). Given the central role of iNOS to induce NO production in response to pro-inflammatory stimuli [10], our study suggests that BT-PGR may suppress the overproduction of NO by inhibiting the expression of iNOS. In alveolar macrophages, it has been reported that NO augments the production of pro-inflammatory cytokines, including TNF-α, IL-1β, and IL-6 [11]. Therefore, we investigated whether the inhibition of NO production by BT-PGR also impacts the production of IL-1β, IL-6, and TNF-α. As illustrated in Fig. 3A, similar to its induction of NO production, the sole treatment with LPS led to an overproduction of IL-1β, IL-6, and TNF-α compared to the untreated cells (Con group). However, treatments with 5% and 10% BT-PGR effectively inhibited the LPS-induced overproduction of these cytokines. Furthermore, our study revealed that treatments with 5% and 10% BT-PGR significantly suppressed the expression of IL-1β, IL-6, and TNF-α (Fig. 3B). These findings suggest that BT-PGR exerts its inhibitory effects by suppressing the expression of IL-1β, IL-6, and TNF-α, thereby preventing their overproduction.

Consequently, the ability of BT-PGR to suppress the production of inflammatory mediators induced by LPS in alveolar macrophages, NR8383 cells underscores its potential as a candidate agent for the prevention of ALI.

### Effect of BT-PGR on MAPK and NF-κB Signaling Pathway in LPS-Stimulated NR8383 Cells

**Fig. 4:**
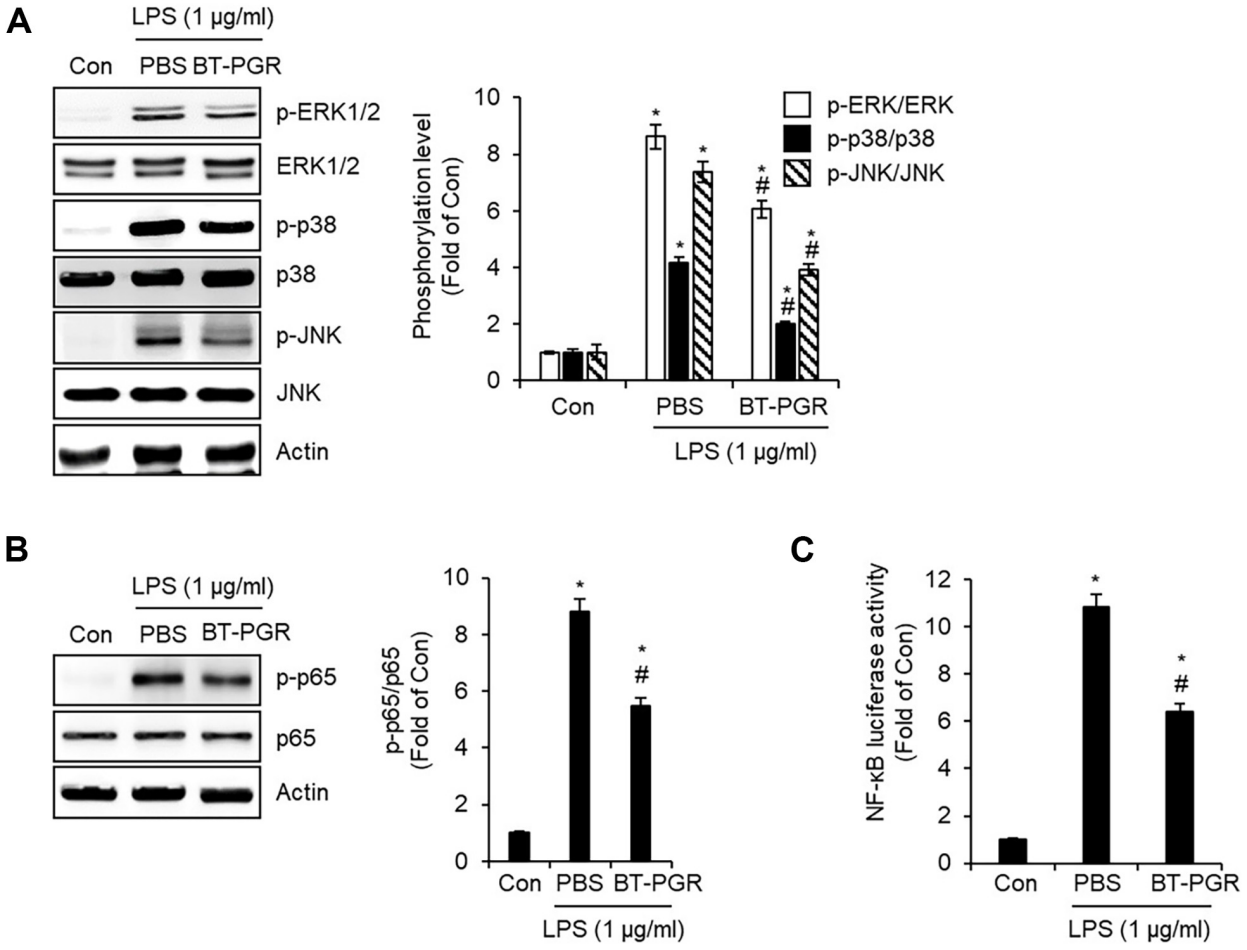
Effect of BT-PGR on MAPK and NF-κB signaling pathways in LPS-stimulated NR8383 cells. NR8383 cells were pretreated with BT-PGR for 2 h and then co-treated with LPS for 30 min. (**A**) The protein level of the MAPK signaling pathway was determined by Western blot analysis. (**B**) The protein level of NF-κB signaling pathways was determined by Western blot analysis. (**C**) NF-κB activity was determined by NF-κB luciferase assay. *P < 0.05 vs. Con group, ^#^*P* < 0.05 vs. PBS group.

It has been reported that the activation of mitogen-activated protein kinases (MAPKs) and nuclear factor kappa B (NF-κB) signaling pathways in macrophage inflammatory responses leads to the overproduction of inflammatory mediators. This process is understood to culminate in the development of chronic inflammation [12, 13]. Thus, we analyzed whether the inhibition of LPS-mediated overproduction of inflammatory mediators by BT-PGR in NR8383 cells is associated with the suppression of MAPK and NF-κB signaling pathways. For this analysis, NR8383 cells were pre-treated with BT-PGR for 2 h followed by a 30-min exposure to LPS. Subsequently, we utilized Western blot analysis to assess the phosphorylation of MAPK signaling components - extracellular signal-regulated kinase 1/2 (ERK1/2), p38, and c-Jun N-terminal kinases (JNK) - as well as the phosphorylation of p65, a key activator in the NF-κB signaling pathway. As shown in Fig. 4A, in comparison with the untreated cells (Con group), exposure to LPS alone elicited the phosphorylation of ERK1/2, p38, and JNK. However, the application of BT-PGR effectively attenuated the LPS-mediated phosphorylation of ERK1/2, p38, and JNK. These findings suggest that BT-PGR inhibits LPS-mediated activation of MAPK signaling pathways in NR8383 cells. Furthermore, we observed that BT-PGR not only significantly reduced the phosphorylation of p65 mediated by LPS but also inhibited the activation of NF-κB luciferase induced by LPS (Fig. 4B and 4C). These results may serve as evidence that BT-PGR effectively inhibits NF-κB signaling pathways. These findings suggest that the inhibition of MAPK and NF-κB signaling pathways by BT-PGR in NR8383 cells contributes significantly to the suppression of the production of inflammatory mediators. However, BT-PGR is a complex mixture of bioactive components, each of which may influence different aspects of the inflammatory response. It remains unclear whether these components act synergistically to regulate inflammation or if specific components are primarily responsible for the observed anti-inflammatory effects. Addressing this issue will require further studies to dissect the contributions of individual components of BT-PGR to the inhibition of key inflammatory signaling pathways.

### Effect of BT-PGR on HO-1 Expression in NR8383 Cells

**Fig. 5:**
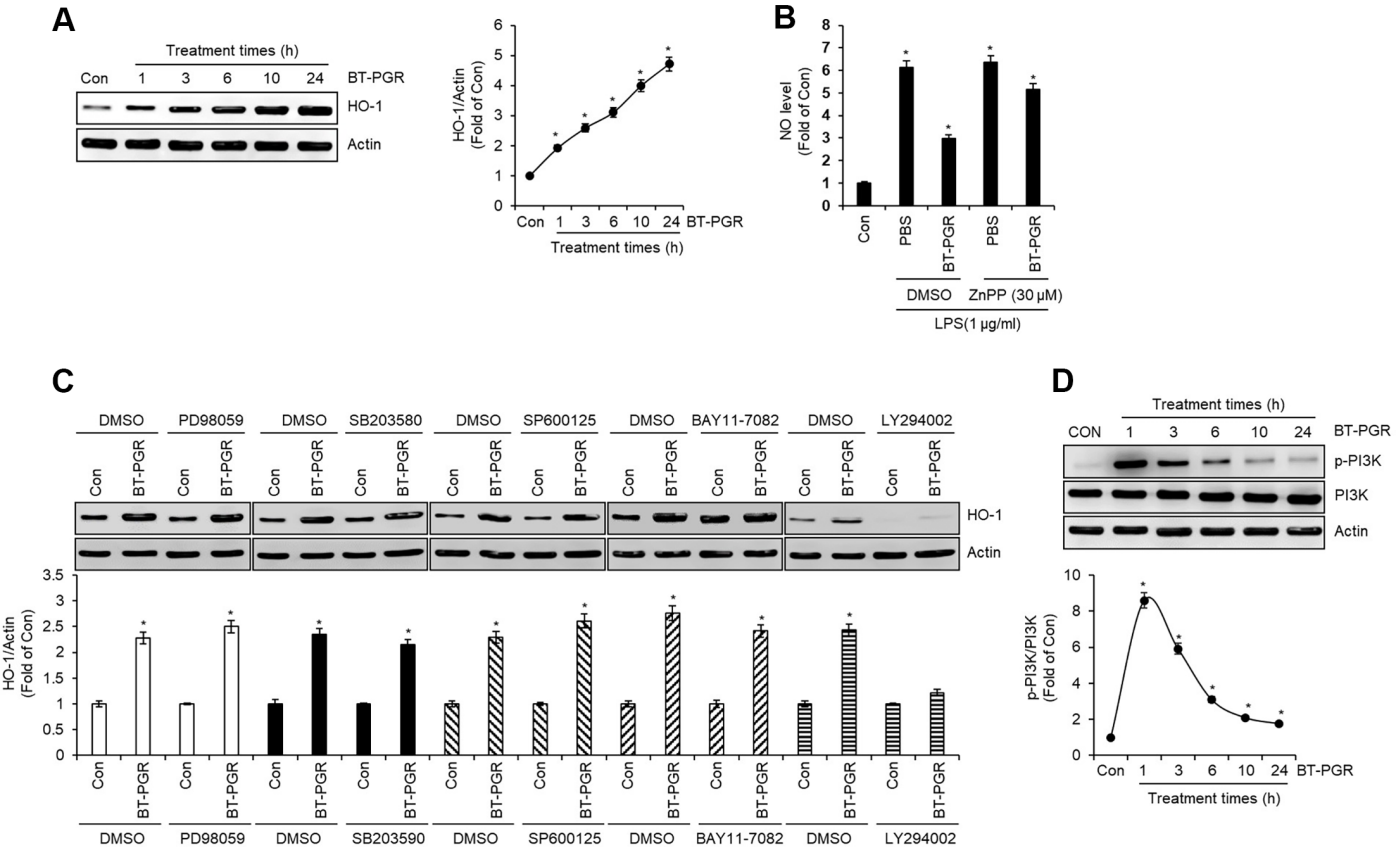
Effect of BT-PGR on HO-1 expression in NR8383 cells. (**A**) NR8383 cells were treated with BT-PGR (5%) for 1 h~24 h. HO-1 level was determined by Western blot analysis. (**B**) NR8383 cells were pretreated with BT-PGR (5%) and ZnPP (30 μM) for 2 h and then co-treated with LPS for 18 h. NO level was determined by Griess assay. (**C**) NR8383 cells were treated with PD98059 (20 μM), SB203580 (20 μM), SP600125 (20 μM), BAY11-7082 (20 μM) or LY294002 (20 μM) and then co-treated with BT-PGR (5%) for 3 h. HO-1 level was determined by Western blot analysis. (**D**) NR8383 cells were treated with BT-PGR (5%) for 1 h~24 h. The levels of p-PI3K and PI3K were determined by Western blot analysis. *P < 0.05 vs. Con group.

Heme oxygenase-1 (HO-1) is recognized as a pivotal antioxidant enzyme, intricately involved in the catabolism of heme into carbon monoxide, iron, and bilirubin. This enzyme plays a critical role in maintaining cellular homeostasis under stress conditions, mediating a protective response against oxidative damage [14]. Among the primary physiological activities of HO-1, its anti-inflammatory action stands out as particularly significant. Consequently, the induction of HO-1 is being employed as a molecular target for the treatment of various human disorders mediated by excessive inflammatory responses [15]. The therapeutic potential of HO-1 induction lies in its capacity to orchestrate a reduction in the production of pro-inflammatory cytokines and other mediators of inflammation, thereby attenuating the inflammatory cascade at multiple levels [16]. Indeed, numerous natural compounds have been reported to effectively induce Heme Oxygenase-1 (HO-1) without eliciting cytotoxic effects [17]. Thus, our investigation focused on determining whether HO-1 plays a role in the suppression of inflammatory mediators induced by BT-PGR. As depicted in Fig. 5A, in NR8383 cells, the expression of HO-1 showed an increase from 1 h following treatment with BT-PGR, in comparison to the untreated cells (Con group). This result demonstrates that BT-PGR can be effectively utilized as a natural inducer for the expression of HO-1. Furthermore, our research extended to investigate whether the induction of HO-1 expression by BT-PGR exerts an influence on the production of the NO. The results indicated that BT-PGR effectively inhibited the LPS-mediated overproduction of NO in the absence of the ZnPP (HO-1 inhibitor) (Fig. 5B). However, when HO-1 was inhibited by ZnPP, BT-PGR only mildly suppressed the LPS-induced excessive NO production (Fig. 5B). These results demonstrate that the expression of HO-1 mediated by BT-PGR contributes to the suppression of inflammatory mediator production induced by BT-PGR.

The expression of HO-1 is induced through the activation of various signaling pathways, with the representative pathways involved being MAPK, NF-κB, and phosphoinositide 3-kinase (PI3K) signaling pathways [18-20]. To investigate the signaling pathways involved in the expression of HO-1 mediated by BT-PGR, we pre-treated NR8386 cells with various signal transduction inhibitors, followed by the administration of BT-PGR. Subsequently, we analyzed the protein levels of HO-1 using Western blot analysis. As illustrated in Fig. 5C, the inhibition of ERK1/2 by PD98059, p38 by SB203580, JNK by SP600125, and NF-kB by BAY11-7082, did not exert any significant impact on the expression of HO-1 mediated by BT-PGR. This finding suggests that these signaling pathways may not be critically involved in the regulation of HO-1 expression by BT-PGR. However, in NR8383 cells where PI3K was inhibited by LY294002, a notable decrease in HO-1 protein expression mediated by BT-PGR was observed compared to cells untreated with LY294002. This significant reduction underscores the potential role of PI3K in the regulation of HO-1 expression by BT-PGR. Thus, to determine whether BT-PGR indeed activates PI3K, we treated NR8383 cells with BT-PGR and subsequently analyzed the phosphorylation of PI3K, indicative of its active form, using Western blot analysis. The results revealed that BT-PGR initiated an increase in the phosphorylation of PI3K starting from 1 h post-treatment. Based on these findings, it is suggested that BT-PGR suppresses the production of inflammatory mediators by inducing the expression of HO-1 in a PI3K-dependent manner.

### Effect of Nrf2 on BT-PGR-Mediated HO-1 Expression in NR8383 Cells

**Fig. 6:**
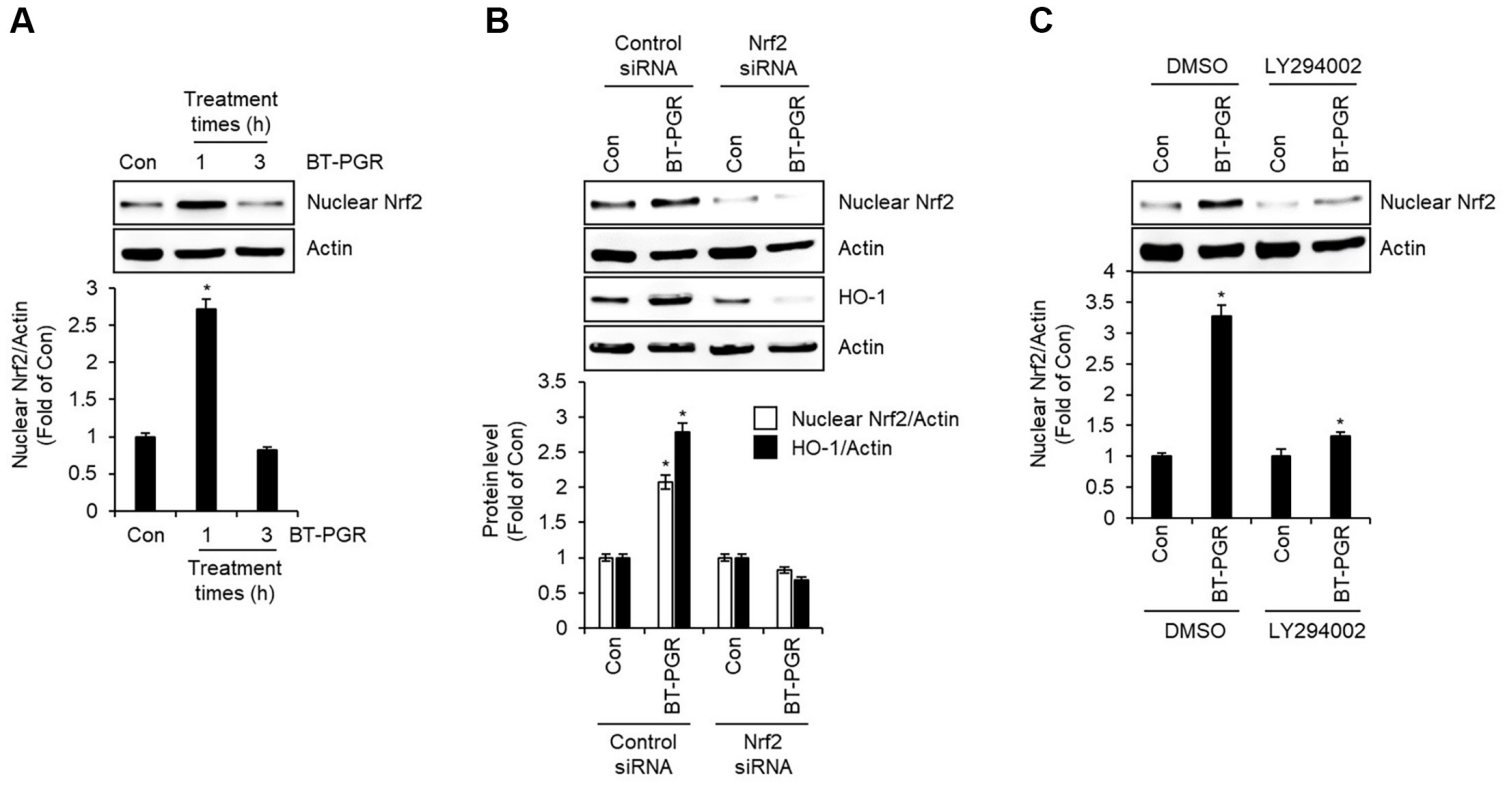
Effect of BT-PGR on nuclear Nrf2 level in NR8383 cells. (**A**) NR8383 cells were treated with BT-PGR (5%) for 1 h~3 h. Nuclear Nrf2 level was determined by Western blot analysis. (**B**) NR8383 cells were transfected with control- or Nrf2 siRNA for 48 h and then treated with BT-PGR (5%) for 1 h. The levels of nuclear Nrf2 and HO-1 protein were determined by Western blot analysis. (**C**) NR8383 cells were treated with LY294002 (20 μM) and then co-treated with BT-PGR (5%) for 1 h. Nuclear Nrf2 level was determined by Western blot analysis. *P < 0.05 vs. Con group.

Nuclear factor erythroid 2-related factor (Nrf2) is considered a multifunctional transcriptional regulator, orchestrating the expression of genes coding for antioxidant, anti-inflammatory, and detoxification proteins [21]. This regulatory role positions Nrf2 as a pivotal factor in cellular defense mechanisms [21]. Among the diverse genes regulated by Nrf2, HO-1 is recognized as a quintessential example [22]. Indeed, numerous studies have reported that the activation of the Nrf2/HO-1 signaling pathway manifests anti-inflammatory activities [23-25]. Thus, to investigate whether the expression of HO-1 mediated by BT-PGR is regulated by Nrf2, we first analyzed the levels of nuclear Nrf2 in BT-PGR-treated NR8383 cells using Western blot analysis. As a result, treatment with BT-PGR led to an increase in the levels of nuclear Nrf2 (Fig. 6A). To investigate whether Nrf2 contributes to the expression of HO-1 mediated by BT-PGR, we conducted a knockdown of Nrf2 using Nrf2 siRNA, followed by treatment with BT-PGR. Subsequently, the levels of BT-PGR-mediated HO-1 protein were analyzed using Western blot analysis. As depicted in Fig. 6B, BT-PGR significantly induced the expression of HO-1 in NR8383 cells where Nrf2 was not knocked down. However, the knockdown of Nrf2 via Nrf2 siRNA resulted in the suppression of BT-PGR-mediated HO-1 expression. Based on these results, it is posited that Nrf2 acts as a potential transcriptional regulator involved in the induction of HO-1 expression mediated by BT-PGR. Furthermore, having confirmed the involvement of PI3K activation in the BT-PGR-mediated expression of HO-1, we evaluated the impact of BT-PGR-mediated PI3K activation on the levels of nuclear Nrf2. As a result, the inhibition of PI3K by LY294002 suppressed the BT-PGR-induced increase in nuclear Nrf2 (Fig. 6C). Considering these results, it is inferred that BT-PGR induces the expression of HO-1 by increasing nuclear Nrf2 in a PI3K-dependent manner.

In this study, we elucidated the anti-inflammatory activity and potential mechanisms of action of deglycosylated PGR (BT-PGR), achieved using the enzyme rapidsase PL Classic derived from *Aspergillus niger*. BT-PGR demonstrated the ability to suppress the production of LPS-mediated inflammatory mediators in the rat alveolar macrophage cell line, NR8383. The suppression of these inflammatory mediators is attributed to the inhibition of LPS-mediated MAPK and NF-κB signaling pathways, as well as the BT-PGR-mediated activation of the PI3K/Nrf2/HO-1 signaling pathway. Based on these results, it can be inferred that BT-PGR exhibits anti-inflammatory activity in rat alveolar macrophages, suggesting its potential as a natural candidate for the inhibition of ALI.

In this study, we used NR8383 cells, which share similar characteristics with alveolar macrophages, to verify the anti-inflammatory activity of BT-PGR. While NR8383 cells are widely employed to model the inflammatory responses of alveolar macrophages, it is challenging to extrapolate the findings from a single cell type to the broader functions of actual alveolar macrophages. Specifically, this study does not account for the complex interactions within lung tissue and among different cell types. Therefore, caution is needed when interpreting the results in the context of alveolar macrophage function in vivo. Future studies should aim to validate these findings in more comprehensive lung tissue models that include alveolar macrophages. In addition, a limitation of this study lies in its reliance solely on in vitro data obtained using NR8383 cells, with a notable absence of in vivo data derived from animal models. Consequently, it is deemed imperative to conduct further in vivo research employing animal models, particularly for the potential clinical application of BT-PGR.

## Figures and Tables

**Table 1:** Sequences of primers used in the amplification of cDNA.

Primers	Sequences
iNOS	Forward 5’-ttgtgcatcgacctaggctggaa-3’
	Reverse 5’-gacctttcgcattagcatggaagc-3’
IL-1β	Forward 5’-ggcaggcagtatcactcatt-3’
	Reverse 5’-cccaaggccacaggtattt-3’
IL-6	Forward 5’-gaggataccactcccaacagacc-3’
	Reverse 5’-aagtgcatcatcgttgttcataca-3’
TNF-α	Forward 5’-tggaactggcagaagaggca-3’
	Reverse 5’-tgctcctccacttggtggtt-3’
GAPDH	Forward 5’-ggactgtggtcatgagcccttcca-3’
	Reverse 5’-actcacggcaaattcaacggcac-3’

**Table 2:** Contents of platycosides in PGR extract before and after biotransformation.

Platycoside	Before biotransformation		After biotransformation	
	Content (%, w/w)	Concentration (mg/ml)	Content (%, w/w)	Concentration (mg/ml)
PE	39.53	1.36	4.27	0.11
PD_3_	3.20	0.11	0.00	0.00
PD	20.06	0.69	22.73	0.59
deapi-PE	12.79	0.44	0.00	0.00
deapi-PD_3_	0.00	0.00	2.93	0.08
deapi-PD	1.16	0.04	25.93	0.67
PGD	18.90	0.65	19.78	0.51
PCAA	4.36	0.15	3.31	0.09
glc-PCD	0.00	0.00	16.76	0.44
glc-PGA	0.00	0.00	2.90	0.08
glc-PCA	0.00	0.00	1.39	0.04
Total	100	3.44	100	2.60
